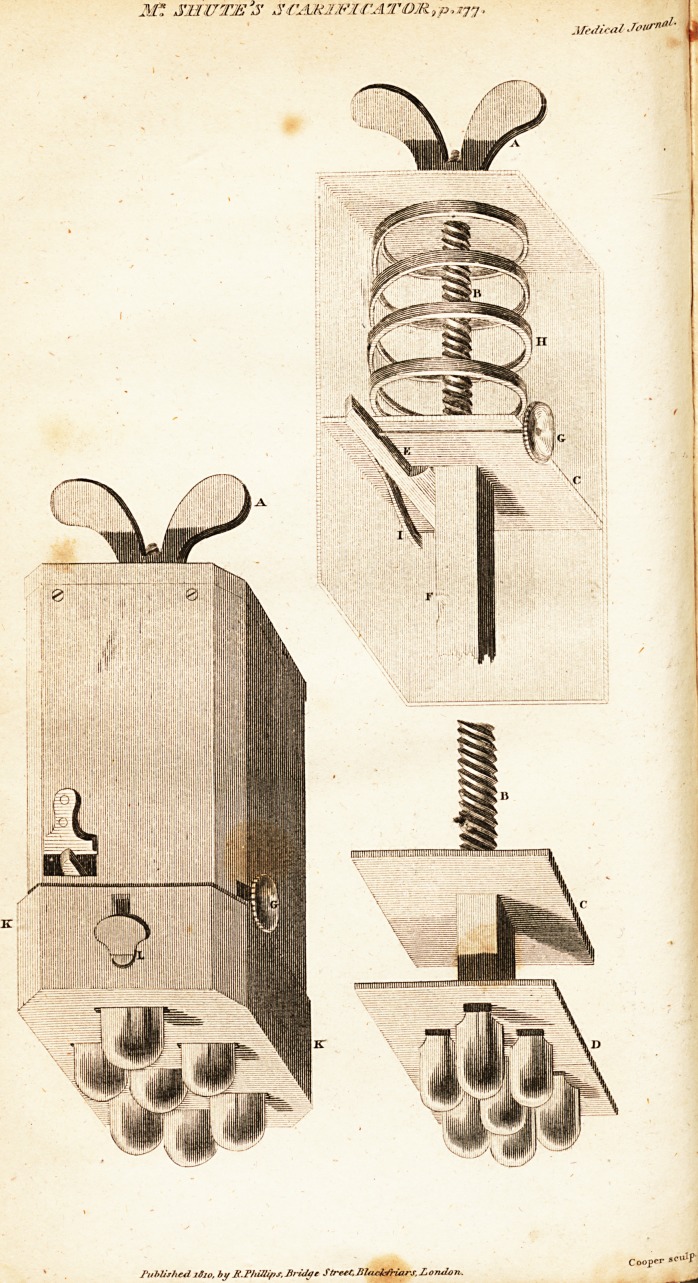# To the Editors of the Medical and Physical Journal

**Published:** 1810-09

**Authors:** Thomas Shute

**Affiliations:** Surgeon. Park Street, Bristol


### Medical and Phyſical Journal.

VOL. XXIV.] SEPTEMBER, 1810. [NO. 139.

*Printed for R. PHILLIPS, by W. Thorne, Red Lion Court, Fleet Street, London.*

### *To the Editors of the Medical and Phyſical Journal.*

GENTLEMEN,

THE advantages resulting from the local evacuation of blood by cupping, in a variety of complaints, being fully established, it would, I presume, be a waste of time elaborately to descant on the merits of such depletion, as forming a high or important remedy in the curative art. It must however be admitted, that the operative means which have been hitherto employed for this purpose, are not only too often tedious and painful in their applications, but very frequently extremely ineffectual in the event. Such being the acknowledged fact, and regarding it as very improbable, that the difficulty of obtaining blood could depend upon a want of manual dexterity in the operator, when the scarificator usually employed had passed through the hands of so many able practitioners, it seemed natural to conclude that the want of success ought rather to be attributed to some fault in the construction of the instrument itself. Impressed with these ideas, and having taken up an opinion, that the failure of the scarificator now in use might be attributed to the manner in which the incisions are made, and supposing that simple punctures would more certainly enter the depth intended, I flatter myself, that by altering the principle upon which the instrument used to act, I have procured one which will effect all the purposes required, with more facility to the operator and less pain to the patient.

Without any intention, then, extravagantly to extol the properties of a new instrument, or unnecessarily to depreciate the merits of an old one, I take the liberty of recommending one to my medical brethren for their approbation, which I have found to answer in my hands much better than any other which I have yet been able to procure.

That the instrument here recommended will invariably produce the wished for effect, I am sanguine enough to believe; at the same time, that I by no means intend to assert that it is not still capable of farther improvement.

A draft taken by Mr. M'Donald, a friend and pupil of mine, is subjoined, sufficiently explanatory, as I hope, of the fabric of the instrument, which may be purchased of Mr. Winter, cutler, Bridge-street.

It is my intention, at no very distant period, to offer a few observations on the formation and number of the lancets, so as more immediately to adapt them to particular parts of the body.

I am, &c.

THOMAS SHUTE, Surgeon.

*Park Street, Bristol,*

*July* 19, 1810.

EXPLANATION of the PLATE.

A. A nut, by means of which, acting on the screw B, the plates C and D holding the lancets, are drawn upwards till the catch E falls into the notch at F. The nut is then unscrewed, and by touching the knob G the catch is withdrawn, and the worm spring H forces the lancets immediately down. I. A spring acting upon the catch E. K. A box which, by means of the screws L L, regulates at will the exposure of the lancets, and in consequence, the depth of the incisions.

## Figures and Tables

**Figure f1:**